# The role of the light chain in the structure and binding activity of two cattle antibodies that neutralize bovine respiratory syncytial virus

**DOI:** 10.1016/j.molimm.2019.04.026

**Published:** 2019-08

**Authors:** Jingshan Ren, Joanne E. Nettleship, Gemma Harris, William Mwangi, Nahid Rhaman, Clare Grant, Abhay Kotecha, Elizabeth Fry, Bryan Charleston, David I. Stuart, John Hammond, Raymond J. Owens

**Affiliations:** a The Division of Structural Biology, Henry Wellcome Building for Genomic Medicine, Roosevelt Drive, Oxford, OX3 7BN, UK; b Research Complex at Harwell, R92 Rutherford Appleton Laboratory, Didcot, OX11 0FA, UK; c The Pirbright Institute, Ash Road, Pirbright, Woking, GU24 0NF, UK

**Keywords:** VH, heavy chain variable domain, VL, light chain variable domain, CDR, complementarity determining region, bRSV, bovine respiratory syncytial virus, H3, CDR3 of the heavy chain variable domain, LC, light chain, HC, heavy chain, Respiratory syncytial virus (RSV), Bovine, Variable domains, Antibody

## Abstract

Cattle antibodies have unusually long CDR3 loops in their heavy chains (HCs), and limited light chain (LC) diversity, raising the question of whether these mask the effect of LC variation on antigen recognition. We have investigated the role of the LC in the structure and activity of two neutralizing cattle antibodies (B4 and B13) that bind the F protein of bovine respiratory syncytial virus (bRSV). Recombinant Fab fragments of B4 and B13 bound bRSV infected cells and showed similar affinities for purified bRSV F protein. Exchanging the LCs between the Fab fragments produced hybrid Fabs: B13* (B13 HC/B4 LC) and B4* (B4 HC/B13 LC). The affinity of B13* to the F protein was found to be two-fold lower than B13 whilst the binding affinity of B4* was reduced at least a hundred-fold compared to B4 such that it no longer bound to bRSV infected cells. Comparison of the structures of B4 and B13 with their LC exchanged counterparts B4* and B13* showed that paratope of the HC variable domain (VH) of B4 was disrupted on pairing with the B13 LC, consistent with the loss of binding activity. By contrast, B13 H3 adopts a similar conformation when paired with either B13 or B4 LCs. These observations confirm the expected key role of the extended H3 loop in antigen-binding by cattle antibodies but also show that the quaternary LC/HC subunit interaction can be crucial for its presentation and thus the LC variable domain (VL) is also important for antigen recognition.

## Highlights

• The Fab structures of two cattle antibodies (B4 and B13) that neutralise bRSV have been solved.
• The light chain plays a critical role in the folding and positioning of CDR H3 of the heavy chains.
• The H3 loop plays a dominant role in antigen-binding.

## Graphical abstract

**Figure:**
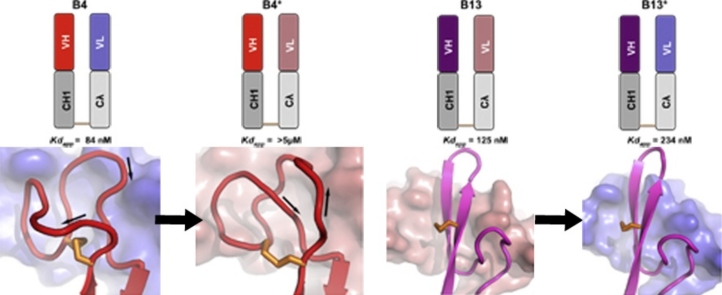

## 1 Introduction

In comparison to human and mice, germ-line immunoglobulin variable region gene diversity in cattle is highly limited. The antibody repertoire is derived from a single polymorphic VH gene family (Berens et al., 1997) and is dominated by one of two VL gene families (Vλ1) (Chen et al., 2008a). Similar to sheep and horses, 90% of the light chains that are expressed in cattle are the λ-isotype. It is generally agreed that V region diversification in cattle is generated by somatic hyper mutation following VDJ segment rearrangement (Verma and Aitken, 2012). The limited sequence diversity in the expressed Vλ repertoire in cattle has led to the suggestion that it contributes relatively little to antigen recognition and that most of the immune response resides in the VH (Saini et al., 2003). A unique feature of the antibody response in cattle is the generation of a subset (up to 10%) of heavy chains that have a highly extended H3 of over sixty residues. This compares to an average of 20 residues for most bovine heavy chains, which in itself is longer than in other species such as human and mouse, typically 8–16 residues. The ultra-long H3s contain a large number of cysteine residues that cross-link to stabilise the structure (Saini et al., 1999; Wang et al., 2013).

We have investigated the role of the light chain in the antigen-binding activity of two bovine monoclonal antibodies (mAbs) by analysing the functional and structural consequences of exchanging their light chains. The two antibodies, designated B4 and B13, previously produced from bovine x mouse heterohybridomas (Taylor et al., 1992; Saini et al., 2003) are specific for the fusion (F) protein of respiratory syncytial virus (RSV). The F protein epitopes recognised by mAbs B4 and B13 are conserved in both human (h) and bovine (b) RSV, and are a major cause of lower respiratory tract infections in young children and calves, respectively. Both B4 and B13 are potent neutralizing, fusion-inhibiting antibodies and protect mice against hRSV infection and calves against bRSV infection (Kennedy et al., 1988; Thomas et al., 1998). Competitive binding assays, recognition of antibody-escape mutants, and binding to synthetic peptides have shown that mAb B4 and B13 recognise different antigenic sites on the F protein. B4 recognises antigenic site II (residues 255–275) (Arbiza et al., 1992; Taylor et al., 1992), whereas B13 binds to an epitope contained within antigenic site IV, V, VI (residues 417–438) (Whyte, 1994). We have produced recombinant Fab fragments of B4 and B13 and also molecules where the heavy and light chains have been exchanged between the two molecules. Analysis of their structures and antigen binding properties suggests a dominant role for the bovine H3 in antigen-binding but shows that the VL also plays a key role in both the folding and positioning of H3 where the functional consequences of this depend on the structure of H3.

## 2 Materials and methods

### 2.1 Protein production

Two vectors were constructed containing resident bovine Cλ and IgG1 CH1 sequences and a signal sequence. The light chain constant region is *Bos taurus* allotypic variant IGLC3a (Genbank DQ537487: and HQ456934; (Chen et al., 2008b)). The CH1 heavy chain is *Bos taurus* IgG1 (Genbank S82409: (Kacskovics and Butler, 1996)). Synthetic genes encoding the constant regions were inserted by Infusion^®^ cloning into PmeI-HindIII cut pOPING-ET (Nettleship et al., 2008b, 2012) The vectors have been engineered so that VH and VL sequences can be inserted into the KpnI-PstI (pOPINBOVL) and KpnI-SfoI (pOPINBOVH) restriction sites by Infusion^®^ cloning. Synthetic genes encoding the candidate variable regions (B4 and B13 VH and VL) (Armour et al., 1994) were purchased from IDT Technology as “Infusion-ready” gBlocks and inserted into the pOPIN expression vectors. All vectors were sequenced to confirm clones were correct. Recombinant Fabs were produced by co-transfection of VH and VL vectors into Expi293™ cells according to the manufacturer’s protocol (Invitrogen). Proteins were purified from culture supernatants by a combination of immobilised metal affinity and size-exclusion chromatography using an automated protocol implemented on an ÄKTAxpress (GE Healthcare) (Nettleship et al., 2009). Purified proteins were analysed by size-exclusion multi-angle light scattering (SEC-MALS) using a Superdex 200 10/300 Increase column (GE Healthcare) and an AktaPure 25 System (GE Healthcare). The protein sample (100 μL), at a concentration of 1.0 mg/mL, was loaded onto the gel filtration column and eluted with one column volume (24 mL) of buffer, at a flow rate of 0.7 mL/min. The eluting protein was monitored using a DAWN HELEOS-II 18-angle light scattering detector (Wyatt Technologies) equipped with a WyattQELS dynamic light scattering module, a U9-M UV/Vis detector (GE Healthcare), and an Optilab T-rEX refractive index monitor (Wyatt Technologies). Data were analysed by using Astra (Wyatt Technologies) using a refractive increment value of 0.185 mL/g. Purified proteins were also analysed under reducing and non-reducing conditions by electrospray mass spectrometry using a Waters Q-Tof Micro mass spectrometer (Nettleship et al., 2008a). Recombinant bRSV F protein was a generous gift from Peter Kwong (National Institutes of Health, Bethesda, MD) (Zhang et al., 2017).

### 2.2 Thermal shift assay

A thermal shift assay (Nettleship et al., 2008a), using the fluorescent dye SYPRO Orange (Invitrogen), was employed to determine protein stability. Solutions of Fabs at 4 μM and 1000x SYPRO orange were made in 20 mM Tris pH 7.5, 200 mM NaCl and 40 μl was added to a 96-well thin-wall PCR plate (Thermo Scientific). The plates were sealed with adhesive PCR seal (4titude) and heated in an Mx3005p qPCR machine (Stratagene) from 25 to 95 °C at a rate of 1 °C/min. Fluorescence changes were monitored with excitation and emission wavelengths of 492 and 610 nm respectively.

### 2.3 Antigen-binding assays

An antigen-binding ELISA assay was performed as previously described (Taylor et al., 1995). Briefly, 50 μl of virus antigen (lysate from BRSV infected Vero cells) was coated onto immunoassay plates alongside control antigen (lysate from mock-infected Vero cells) and left to dry overnight at 37 °C. The wells were then blocked for 1 h at room temperature with 200 μl PBS containing 5% pig serum and 0.05% Tween 20. After washing the wells four times with PBS containing 0.05% Tween 20, serial dilutions of Fabs were added to the wells (final volume of 50 μl in each well) followed by incubation at room temperature for 1 h. The wells were washed five times with PBS containing 0.05% Tween 20 and 50 μl 1:4000 Rabbit anti-Bovine IgG F(ab′)2HRP (Sigma SAB3700010) conjugate added to each well. The plate was incubated at room temperature for 1 h. After washing five times in PBS containing 0.05% Tween 20, 100 μl freshly prepared TMB substrate was added to each well. The reaction was stopped with 50 μl 1 M H_2_SO_4_ when the colour change had reached the optimum. Absorbance was read at 450 nm and 690 nm.

### 2.4 Surface plasmon resonance

The surface plasmon resonance experiments were performed using a BiaCore T200 (GE Healthcare) equipped with a Series S CM5 sensor chip. The F protein was immobilized using amine-coupling chemistry. The surfaces of the sample and reference flow cells were activated for 7 min with a 1:1 mixture of 0.1 M NHS (*N*-hydroxysuccinimide) and 0.1 M EDC (3-(*N,N-*dimethylamino) propyl*-N-*ethylcarbodiimide) at a flow rate of 5 μL/min. The F protein, at a concentration of 6.7–20 μg/mL in PBS pH 6.5, was immobilized at a density of 20–100 RU on the sample flow cell. The reference flow cell was left blank. All the surfaces were blocked with a 7 min injection of 1 M ethanolamine, pH 8.0.

To collect steady-state affinity data, the Fabs were injected over the two flow cells at a range of concentrations prepared by serial four-fold dilution of the Fab stock from 5 μM (including five duplicates and zero concentration samples), at a flow rate of 30 μL/min and at a temperature of 20 °C. For B4 and B4* the assay was performed in PBS pH 7.4, the complex was allowed to associate and dissociate for 180 and 600 s, respectively, and surfaces were regenerated with two 120 s injections of 0.1 M Glycine pH 3.0, 0.5 M NaCl. For B13 and B13* the assay was performed in PBS pH 7.4 with 0.5% (v/v) surfactant P20 to reduce non-specific binding, the complex was allowed to associate and dissociate for 300 and 300 s, respectively, and surfaces were regenerated with one 120 s injection of 0.1 M Glycine pH 1.5. The data were fitted to a 1:1 interaction steady-state binding model using the BiaEvaluation 1.0 software.

### 2.5 Crystallization, data collection and structure determination

Protein crystallizations were carried out using standard OPPF protocols (Walter et al., 2005). Crystals were grown by the sitting drop vapor diffusion method at room temperature. Each Fab crystallized in different buffer conditions containing PEG 3350 or PEG ME 2000 (see Table S1 for details). All crystals were flash-frozen in liquid nitrogen and then kept at −173 °C under a stream of nitrogen gas during data collection. X-ray data were collected on Diamond beamlines I03 for B4 and B13*, I04-1 for B13 and I24 for B4* Fabs (Diamond Light Source, Harwell, UK), see Table 1.

**Table 1:** Data collection and refinement statistics.

	B4	B4^a^	B13	B13^a^
**Data collection**				
Space group	P21212	P21	P21	P1
Cell dimensions				
*a*, *b*, *c* (Å)	78.4, 135.1, 42.1	42.4, 86.0, 165.0	87.9, 132.5, 118.2	43.4, 76.3, 130.0
α, β, γ (°)	90.0, 90.0, 90.0	90.0, 98.6, 90.0	90.0, 102.9, 90.0	89.7, 87.4, 88.6
Resolution (Å)	67.8–1.90 (1.95–1.90)^a^	76.1–2.15 (2.29–2.15)	85.7–2.12 (2.16–2.12)	65.9–2.62 (2.67–2.62)
*R*_merge_	0.116 (0.922)	0.094 (–)	0.128 (–)	0.044 (0.782)
*I* / σ*I*	14.4 (2.2)	10.3 (1.0)	8.6 (1.8)	13.8 (1.4)
Completeness (%)	99.2(98.0)	95.7 (72.4)	99.2 (97.6)	98.2 (98.1)
Redundancy	23.5 (10.9)	6.1 (3.7)	6.8 (6.0)	3.5 (3.6)
CC_1/2_	–/–b	1.0 (0.55)	1.0 (0.58)	1.0 (0.52)
**Refinement**				
Resolution (Å)	67.7–1.92	76.1–2.15	85.7–2.12	65.9–2.62
No. reflections	33138 (1723)	58184 (2912)	140485 (7327)	46980 (2230)
*R*_work_/*R*_free_	0.229/0.277	0.213/0.235	0.256/0.276	0.237/0.274
No. atoms:				
Protein	2943	6452	19565	13125
Water	59	321	1396	53
*B*-factors:				
Protein	75	59	23	96
Water	53	51	39	60
R.m.s. deviations:				
Bond lengths (Å)	0.016	0.005	0.010	0.012
Bond angles (°)	1.8	0.8	1.4	1.6

a Values in parentheses are for highest-resolution shell.

b CC50 was not incorporated in the data processing programs used for this data set.

The diffraction data were indexed, integrated and merged with the automatic data processing program Xia2 using the 3dii or Dials protocols (Winter, 2010; Waterman et al., 2016). The statistics of the data are shown in Table 1. The structure of the B4 Fab was solved first by molecular replacement using the coordinates of a human Fab, FabOX108 (PDB ID, 3DGG) as the search model. The orientation and position of the model in the crystal were determined using the program MOLREP (Vagin and Teplyakov, 1997) and refined with rigid-body refinement followed by restrained maximum-likelihood positional and B-factor refinements, using either REFMAC (Murshudov et al., 1997) or PHENIX (Janowski et al., 2016). The refined structure of the B4 Fab was then used as a starting model for the structure determinations of the three remaining Fabs. Model rebuilding was carried out with COOT (Emsley et al., 2010).

## 3 Results

### 3.1 Expression and purification of recombinant Fabs

Cloning of the heavy and light chain variable regions of the anti-bRSV bovine monoclonal antibodies (B4 and B13) has been previously reported (Armour et al., 1994) and this information was used to construct recombinant Fabs of the two antibodies. Synthetic genes encoding the variable regions were inserted into plasmids containing resident signal sequences and light and heavy chain constant regions to produce B4 and B13 Cλ LC and IgG1Fd HC expression vectors respectively. A hexahistidine tag was added to the C-terminus of the HC for detection and separation of assembled Fabs from LC dimers.

**Fig. 1:**
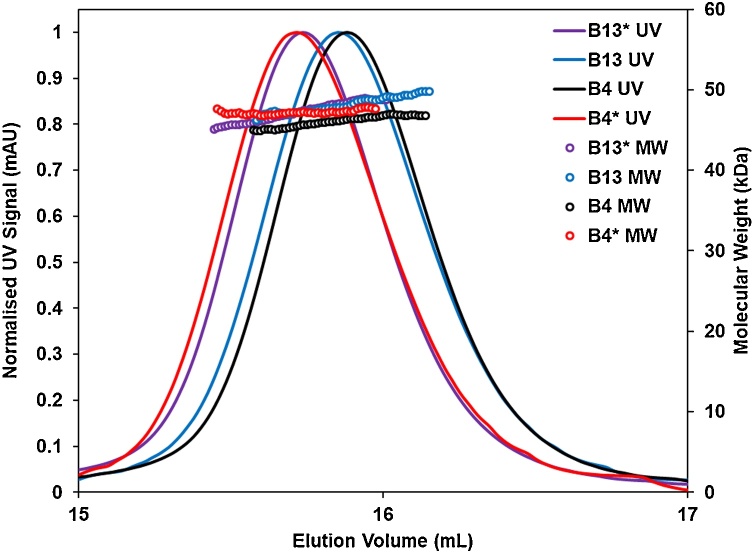
SEC-MALS profiles for the anti-RSV Fabs showing expected molecular weights for the intact Fabs with the inter-molecular disulphide bond intact. The results showed the molecular weight of B4 to be 46 kDa (calculated 47.7 kDa); B13 to be 48 kDa (calculated 48.4 kDa); B4* to be 46 kDa (calculated 47.7 kDa) and B13* to be 48 kDa (calculated 48.4 kDa)(For colour coding of SEC-MALS profiles in this figure , the reader is referred to the web version of this article).

**Fig. 2:**
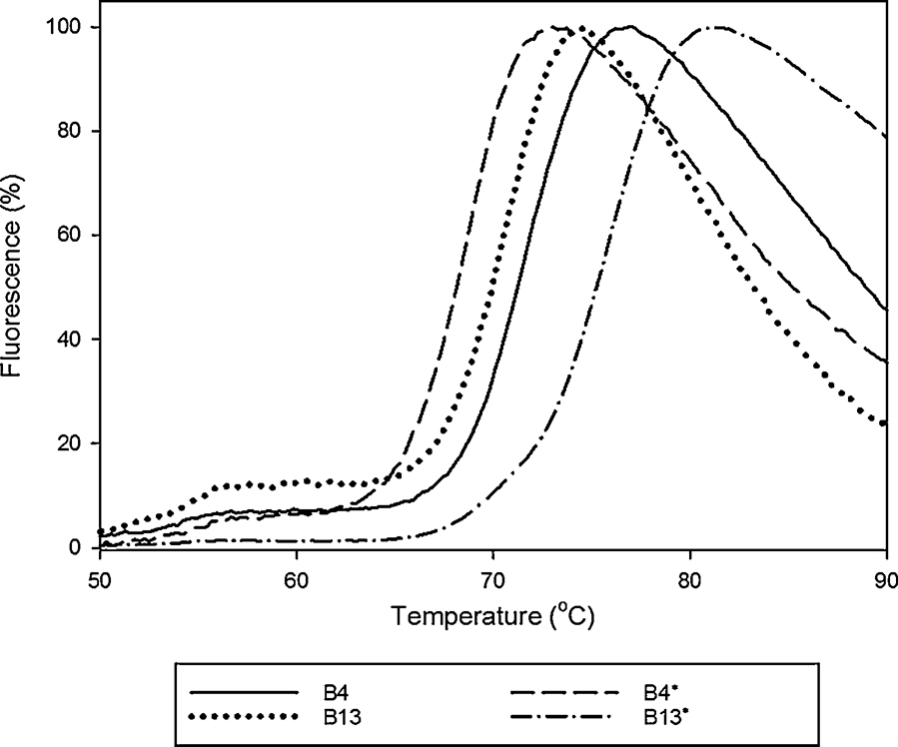
Melt curves from the thermal shift assay on all four Fabs showing high stability for both the matched and the chain-swapped versions. The *T*_m_ values for each Fab are: 71 ± 1 °C for B4; 70 ± 1 °C for B13; 68 ± 1 °C for B4* and 75 ± 1 °C for B13*.

To investigate the role of the LCs in the assembly of the Fabs, Expi293™ cells were co-transfected with all combinations of light and heavy chains to produce four Fabs B4, B4*, B13 and B13*. Transfection of HEK cells with either B4 and B13 HC alone did not produce any heavy chain only antibody fragments confirming that assembly with the LC was essential for production of secreted protein. The secreted products were purified by IMAC followed by gel filtration and purity confirmed by mass spectrometry. Assembly of the recombinant B4, B13 Fd HC fragments with either B4 or B13 LCs into Fab fragments was demonstrated by SEC-MALS run under non-reducing conditions with the molecular weights of all four proteins corresponding to the expected size for a Fd-LC dimer (Fig. 1). The thermal stability of the assembled Fabs was probed using a Thermal Shift Assay (Nettleship et al., 2008a) and all, including the LC swapped Fabs, were found to be very stable (Fig. 2). The transition melting temperatures (*T_m_*) for the B4 and B13 Fabs and the hybrid B4* were within 1 °C of each other whereas the B13* showed a 5 °C +/- 1 °C increase in *T_m_* indicating a stronger association of heavy and light chains.

### 3.2 Antigen-binding activity

**Fig. 3:**
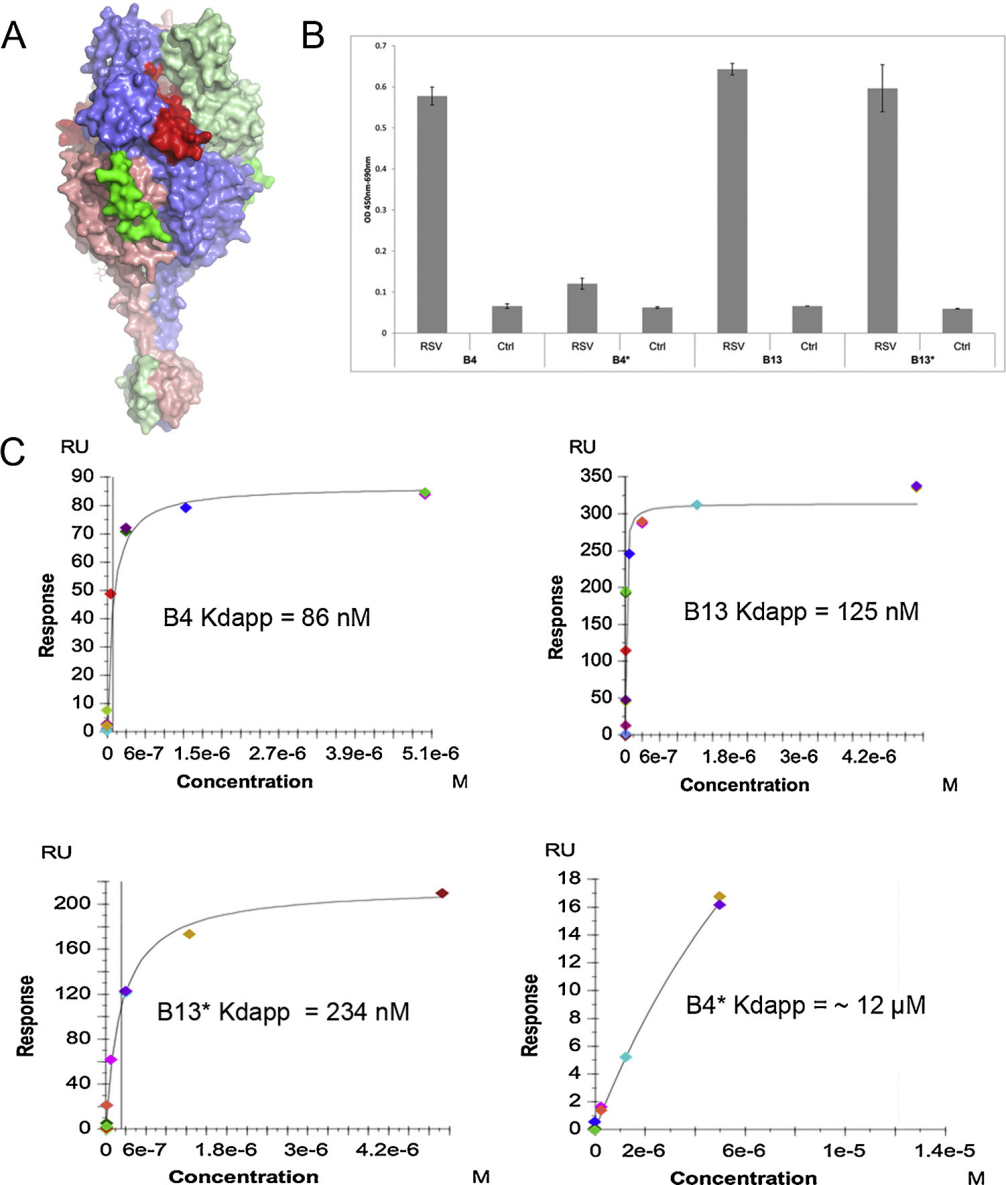
(A) Surface representation of bovine RSV F trimer (PDB code 5TDG)^27^. 3 monomers are coloured in light blue, pale green and salmon, respectively. The antigenic site for B4 is in red and B13 in green. (B) ELISA data showing binding of Fabs to bRSV antigen against a negative control antigen. (C) SPR data fitted to a 1:1 steady-state affinity model. The vertical black lines indicate the *K_dapp_* obtained from the fit, with the exception of B4* where this is shown by a dashed red line. (For interpretation of the references to colour in this figure legend, the reader is referred to the web version of this article.)

The location of the epitopes recognized by B4 and B13 were mapped onto the structure of the bRSV F protein to illustrate that their binding sites are spatially separate (Fig. 3a). The recombinant Fabs were assessed for binding to bovine RSV (bRSV) in an antigen-binding ELISA using lysed Vero cells infected with bRSV as the source of antigen and the lysate of mock-infected cells as the control. Fig. 3b shows that both the matched Fabs (B4 and B13) and one of the hybrid Fabs, B13*, showed similar binding, whereas the B4* light chain-swapped Fab showed a considerably reduced binding activity that was not significantly different from the mock control.

Detailed analysis of antigen-binding to purified bRSV F protein was carried out by Surface Plasmon Resonance (SPR). The F protein was immobilised on the surface of the sensor chip and the Fabs passed over the surface. The assays were initially conducted in PBS pH 7.4 but it was found that B13 and B13* displayed a high proportion of non-specific binding. Therefore, the assay conditions were extensively optimised for these two Fabs to reduce the effects of non-specific binding. The sensogram data were fitted to a 1:1 steady-state binding model under the assumptions that each subunit of the trimeric F protein binds one Fab, and that each subunit binds the Fab with the same affinity i.e. that there is no cooperativity between the F protein subunits upon Fab binding. These assumptions, and the presence of an element of non-specific binding, mean that the *K_d_*s obtained from this analysis should only be treated as apparent *K_d_*s. The *K_dapp_* obtained for the F protein/B4, B13 and B13* were 84 nM, 125 nM and 234 nM respectively (Fig. 3c). For B4* an accurate *K_dapp_* could not be calculated as the assay had not reached saturation at a Fab concentration of 5 μM which was the maximum B4* concentration used in the assay, but suggests that the affinity would be in the micromolar range (Fig. 3b). Overall, the results of binding to purified F protein are consistent with the results of the cell lysate ELISA and demonstrate that B4* shows much weaker binding to the F protein than the other Fabs.

### 3.3 Overall structure of Fabs

**Fig. 4:**
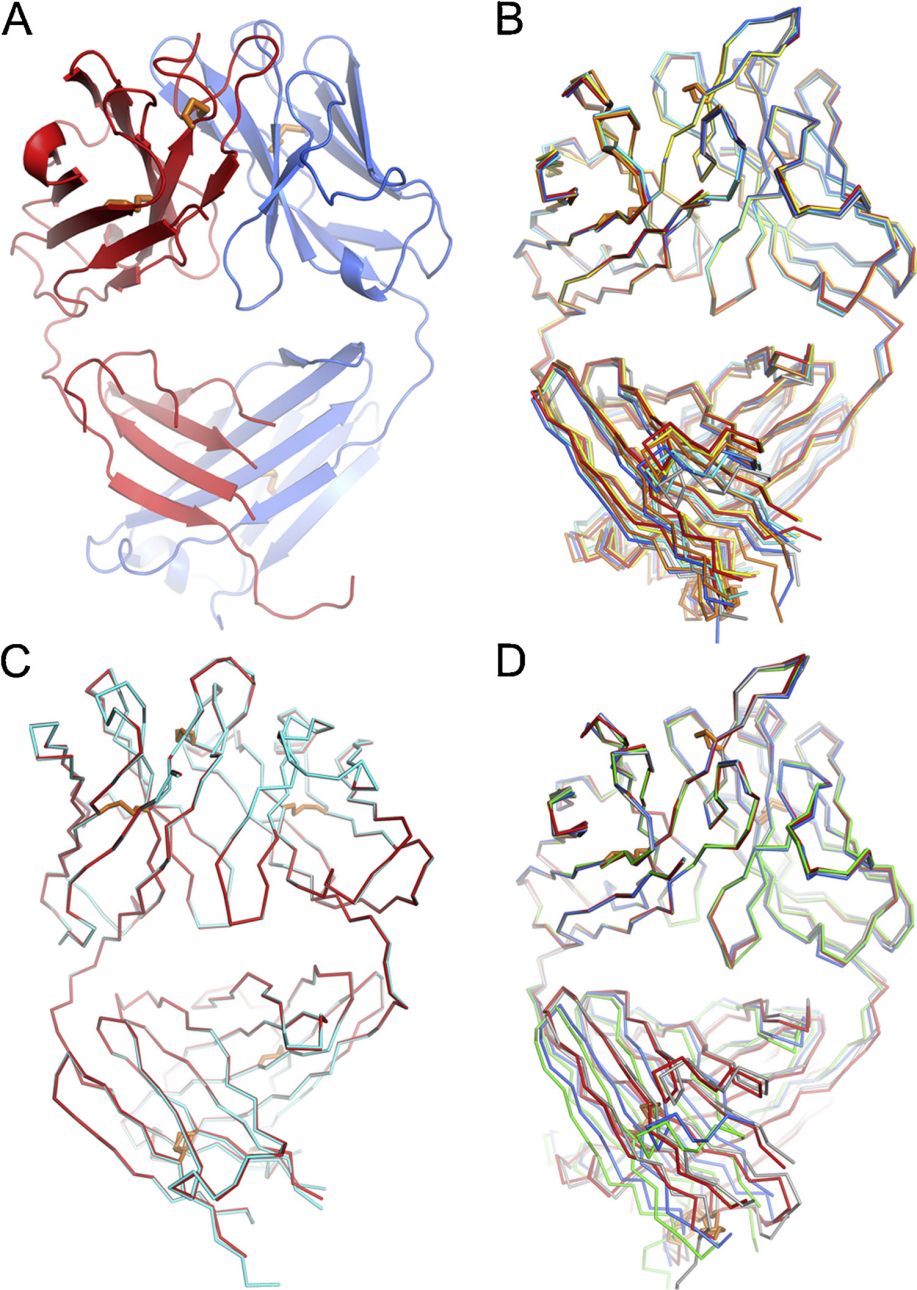
Overall structures of 4 Fabs. (A) B4 with HC in red and LC in blue. (B)-(D) Overlaps of 6 B13 (B), 2 B4* (C) and 4 B13* (D) Fabs in the crystallographic asymmetric units. Superimpositions are based on the variable domains only. (For interpretation of the references to colour in this figure legend, the reader is referred to the web version of this article.)

To investigate the structural basis of the different functional effects of exchanging the light chains between B4 and B13, all four Fabs were crystallized, diffraction data collected and their structures solved by molecular replacement using an initial model of human FabOX108 (PDB ID: 3DGG) (Nettleship et al., 2008b) (Table 1). There were 1, 6, 2 and 4 Fab molecules in the crystallographic asymmetric units (AU) of the B4, B13, B4* and B13* crystals, respectively. For B4 a large portion of the CH1 domain of the heavy chain was disordered, including residues 149–153, 169–179, 198–215 and 222−233. For the Fabs with more than one molecule in the AU superimposition showed that the 6 B13 Fabs differed by rmsds of 0.7–1.4 Å, the 2 B4* Fabs by only 0.3 Å and the 4 B13* Fabs by 0.5–2.3 Å based on Cα atoms. The large structural differences in B13 and B13* Fabs are mainly due to variations in the elbow angles between the variable and constant domains. Thus, superimposition of the variable domains of the 6 B13 and 4 B13* Fabs showed smaller conformational differences between the variable domains with rmsds of 0.4−0.6 Å and 0.8−0.9 Å respectively (Fig. 4).

### 3.4 Comparison of the variable domains of B4 and B13

**Fig. 5:**
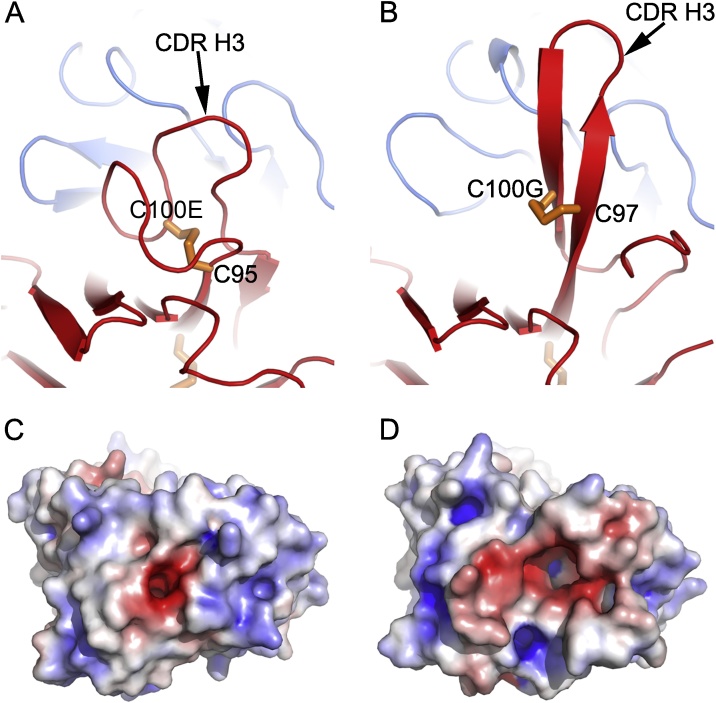
Conformations of CDR H3s. (A) B4 Fab. (B) B13 Fab. In both (A) and (B) HC is coloured in red and LC in blue. The disulphides are shown as orange sticks (note C100E is Kabat numbering). Surface charge profiles of the variable domains of B4 Fab (C) and B13 Fab (D) viewed from the top. (For interpretation of the references to colour in this figure legend, the reader is referred to the web version of this article.)

The H3s of B4 and B13 are of different lengths, comprising 21 and 25 residues respectively. Both contain a pair of cysteines, at positions cys97-cys100G (B4), and cys95-cys100E (B13) (Kabat numbering) (Fig. 5) and structures of the Fabs revealed that they form disulphide bridges irrespective of the paired LC. Both H3s adopt a double loop structure, though with different configurations. The two loops of B4 H3 are of a similar length with the N-terminal loop contacting the CDR3 of the LC (L3) and the C-terminal loop interacting with CDR1 (L1) and CDR2 (L2). The N-terminal loop of the longer B13 H3 adopts an extended β-hairpin structure contacting L1 and L2, and a shorter C-terminal loop folded between the H1 and L2 loops (Fig. 5). Therefore, in contrast to the H3 of B4, that of B13 protrudes from the surface of the variable regions. Viewed from the top, the antigen binding regions of the two Fabs show differences in both shape and electrostatic potential (Fig. 5). Both Fabs have a negatively charged cavity surrounded by positively charged areas, but the B13 cavity is much bigger and the negatively charged area is broader, consistent with the fact that the two Fabs bind to different epitopes of the bRSV F protein (Fig. 3a).

### 3.5 Comparison of light chain exchanged Fabs

**Fig. 6:**
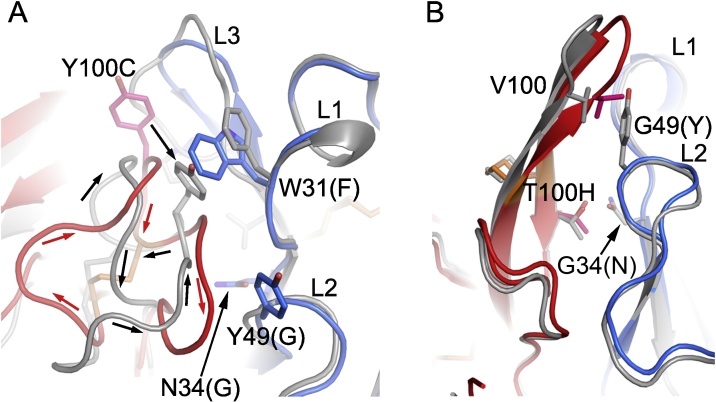
The effects of LC on the structure of HC. (A) Structural differences between B4 H3 and B4* H3. B4 HC is in red and B4 LC in blue, B4* is in grey. The running directions of H3s are indicated by arrows. (B) Structural differences between the H3s of B13 (red and blue) and B13* (grey). Side chains and disulphides are shown as sticks. Residues are shown in Kabat numbering. For the light chains the final letter is in brackets and represents the one letter code for the sequence of the hybrid Fab (in all cases the letter preceding the residue represents the amino acid of the native Fab). (For interpretation of the references to colour in this figure legend, the reader is referred to the web version of this article.)

In order to investigate how light chain pairing may affect the structure of the heavy chain variable domain and hence antigen binding, the structure of B4 was compared with B4*, and B13 with B13*. Interface analysis of the paired variable regions of the heavy and light chains showed little difference for the B13 HC with either LC. Table S1 shows a similar number of residues involved in the interface and similar interface surface area. A detailed analysis shows LC residues 47–52 in the B13* structure are less buried than those in the B13 structure. For example, three residues in the B13* Fab structure are solvent exposed (Ile47, Glu50 and Ser52) whereas they form part of the interface in the B13 structure (Ile47 is 100% buried, Asn50 is 50% buried and Lys 52 is 20% buried) (Table S1). Overlapping the H3 of B13* and B13 shows that the overall β-hairpin structure of the H3 remains unchanged when the B13 LC is replaced by the B4 LC, despite being pushed slightly away by 2.5 Å from the light chain due to the presence of Tyr49 in the B4 LC instead of a glycine (Fig. 6b). The observation that only small structural differences in the B13 HC occur on exchanging the B13 LC for the B4 LC is consistent with the very modest reduction in antigen-binding affinity measured by SPR.

By contrast, swapping the B4 LC for B13 LC leads to large changes in both orientation of the B4 VH domain and conformation of the H3 loop. The VH domain of B4* differs by approximately 10° in orientation relative to the VL domain compared to B4 VH (Fig. 6a). On exchanging the light chain from B4 to B13, the H3 flips around to include a π-interaction between Tyr100C (Kabat numbering) of B4 HC and Phe31 of the CDR1 of B13 LC. In the B4 Fab structure, this space is occupied by Trp31, which does not allow space for the Tyr100C interaction. In addition, the orientation of the H3 loop changes from the sequence running clockwise when viewed from the top in the B4 structure to anticlockwise in the B4* structure (Fig. 6a).

The area of the interface between the variable domains of the B4* Fab is lower than that for the B4 (Table S1). This analysis shows that amino acids 100E to 100 L of the HC in the B4* structure have very little involvement in the interface except for Gly100 G, which is completely buried. In the B4 Fab structure, all of these amino acids are involved in the interface with ≥20% of the amino acid buried (Table S1). This string of amino acids at the interface in the B4 structure contributes four hydrogen bonds, involving Cys100E, Thr100 F, Gly100 G and Glu100 L, which are not present at the B4* interface. The large conformation change that occurs on replacing the B4LC with B13LC radically alters the conformation of H3 and this disruption of the antigen-binding surface is consistent with the loss of activity.

## 4 Discussion

In this study, we have examined the role of light and heavy chain pairing in the antigen-binding activity of two bovine monoclonal antibodies to the F protein of bRSV, B4 and B13. We have analysed the functions and structures of the Fab fragments from both parent antibodies expressed as recombinant proteins and versions of the Fabs in which the light chains had been exchanged. Overall, these results demonstrate that for B4 and B13, binding to the cognate LC is not essential for the formation of a stable Fab fragment. However, although all combinations of light and heavy chain assembled, there were different outcomes in respect of antigen-binding. In the case of B13 there was only a small reduction in binding affinity on swapping with the B4 light chain whereas exchanging B4LC for B13LC significantly compromised antigen-binding affinity by Fab B4. By examining the structures of all four Fabs, these observations could be explained by differences in the role of the LCs in positioning the H3 loops of the B4 and B13. In the case of B13, the conformation of H3 was only marginally affected by pairing with B4 VL, whereas for B4, the conformation of H3 was radically altered. The CDR loops in the B13 and B4 VL domains showed little change in conformation between the parent and chain swapped Fabs pointing to the key role of H3 in antigen-binding.

It has been shown that all CDR loops of antibody variable regions, with the exception of H3, adopt one of a limited number of canonical structures (Chothia and Lesk, 1987) including bovine antibodies (Stanfield et al., 2016), though the number of structures available for analysis is very small compared to human and mouse. Of the six CDRs, H3 shows the greatest structural variability and in comparison with loops matched in length from non-antibody proteins, adopts a high proportion of unique conformations (Regep et al., 2017) and is generally considered to play the major role in antigen-binding interactions in most antibodies.

The pairing of immunoglobulin light and heavy chains contributes to the diversification of antibody binding sites and the stability of the antibody structure (Chatellier et al., 1996). The preference in pairing of particular heavy and light chains was presumed to occur at random (Berens et al., 1997; Brezinschek et al., 1998, de Wildt1999). In humans, LCs from multiple sub-groups can pair with a single IGHV and in several cases use of an IGLV or IGKV combines with identical VDJ combinations without loss of antigen specificity (Edwards et al., 2003). However, pairing preferences have been shown to exist in the human and mouse germline for a small proportion of gene segments, whilst others are promiscuous and show no pairing preferences (Jayaram et al., 2012). Specific pairings in cattle have been shown for the ultra-long H3 in cattle; these ultra-long sequences have unique structural properties and restricted LC pairings (Saini et al., 2003) that may have evolved specifically to provide a structural framework for supporting the ultra-long H3 (Wang et al., 2013). The limited diversity of the cattle VL germ line sequences leads to the hypothesis that the LC repertoire performs a greater structural role than in other species.

In conclusion, the analysis of two bovine antibodies with H3 of more typical length (21–25 residues) presented here supports this view, and shows that the pairing of LC/HC can be crucial for the structure of the antibody paratope. Thus the LC variable domain (VL) is important for antigen recognition, contrary to what might have been expected from the limited VL repertoire in cattle but in line with the co-evolution of these protein domains.

Further work on the structure of the B13 and B4 in complex with the RSV F protein will be required to determine whether this involves any direct interactions between the LC and the antigen.

## Accession numbers

The atomic coordinates and structure factors of the Fabs have been deposited in the Protein Data Bank under the accession codes 6QN9, 4QN8, 6QN7 and 6QNA for B4, B13, B4* and B13* respectively.

## References

[bib0005] Arbiza J., Taylor G. (1992). Characterization of two antigenic sites recognized by neutralizing monoclonal antibodies directed against the fusion glycoprotein of human respiratory syncytial virus. J. Gen. Virol..

[bib0010] Armour K.L., Tempest P.R. (1994). Sequences of heavy and light chain variable regions from four bovine immunoglobulins. Mol. Immunol..

[bib0015] Berens S.J., Wylie D.E., Lopez O.J. (1997). Use of a single VH family and long CDR3s in the variable region of cattle Ig heavy chains. Int. Immunol..

[bib0020] Brezinschek H.P., Foster S.J. (1998). Pairing of variable heavy and variable kappa chains in individual naive and memory B cells. J. Immunol..

[bib0025] Chatellier J., Van Regenmortel M.H. (1996). Functional mapping of conserved residues located at the VL and VH domain interface of a Fab. J. Mol. Biol..

[bib0030] Chen L., Li M. (2008). Characterization of the bovine immunoglobulin lambda light chain constant IGLC genes. Vet. Immunol. Immunopathol..

[bib0035] Chen L., Li M., Li Q., Yang X., An X. (2008). Characterization of the bovine immunoglobulin lambda light chain constant IGLC genes. Vet. Immunol. Immunopathol..

[bib0040] Chothia C., Lesk A.M. (1987). Canonical structures for the hypervariable regions of immunoglobulins. J. Mol. Biol..

[bib0045] de Wildt R.M., Hoet R.M. (1999). Analysis of heavy and light chain pairings indicates that receptor editing shapes the human antibody repertoire. J. Mol. Biol..

[bib0050] Edwards B.M., Barash S.C. (2003). The remarkable flexibility of the human antibody repertoire; isolation of over one thousand different antibodies to a single protein, BLyS. J. Mol. Biol..

[bib0055] Emsley P., Lohkamp B. (2010). Features and development of Coot. Acta Crystallogr. D: Biol. Crystallogr..

[bib0060] Janowski P.A., Moriarty N.W. (2016). Improved ligand geometries in crystallographic refinement using AFITT in PHENIX. Acta Crystallogr. D: Struct. Biol..

[bib0065] Jayaram N., Bhowmick P. (2012). Germline VH/VL pairing in antibodies. Protein Eng. Des. Sel..

[bib0070] Kacskovics I., Butler J.E. (1996). The heterogeneity of bovine IgG2--VIII. The complete cDNA sequence of bovine IgG2a (A2) and an IgG1. Mol. Immunol..

[bib0075] Kennedy H.E., Jones B.V. (1988). Production and characterization of bovine monoclonal antibodies to respiratory syncytial virus. J. Gen. Virol..

[bib0080] Murshudov G.N., Vagin A.A. (1997). Refinement of macromolecular structures by the maximum-likelihood method. Acta Crystallogr. D: Biol. Crystallogr..

[bib0085] Nettleship J.E., Brown J. (2008). Methods for protein characterization by mass spectrometry, thermal shift (ThermoFluor) assay, and multiangle or static light scattering. Methods Mol. Biol..

[bib0090] Nettleship J.E., Ren J. (2008). A pipeline for the production of antibody fragments for structural studies using transient expression in HEK 293T cells. Protein Expr. Purif..

[bib0095] Nettleship J.E., Rahman-Huq N. (2009). The production of glycoproteins by transient expression in mammalian cells. Methods Mol. Biol..

[bib0100] Nettleship J.E., Flanagan A. (2012). Converting monoclonal antibodies into Fab fragments for transient expression in mammalian cells. Methods Mol. Biol..

[bib0105] Regep C., Georges G. (2017). The H3 loop of antibodies shows unique structural characteristics. Proteins.

[bib0110] Saini S.S., Allore B. (1999). Exceptionally long CDR3H region with multiple cysteine residues in functional bovine IgM antibodies. Eur. J. Immunol..

[bib0115] Saini S.S., Farrugia W. (2003). Bovine IgM antibodies with exceptionally long complementarity-determining region 3 of the heavy chain share unique structural properties conferring restricted VH + Vlambda pairings. Int. Immunol..

[bib0120] Stanfield R.L., Wilson I.A. (2016). Conservation and diversity in the ultralong third heavy-chain complementarity-determining region of bovine antibodies. Sci. Immunol..

[bib0125] Taylor G., Stott E.J. (1992). Protective epitopes on the fusion protein of respiratory syncytial virus recognized by murine and bovine monoclonal antibodies. J. Gen. Virol..

[bib0130] Taylor G., Thomas L.H. (1995). Role of T-lymphocyte subsets in recovery from respiratory syncytial virus infection in calves. J. Virol..

[bib0135] Thomas L.H., Cook R.S. (1998). Passive protection of gnotobiotic calves using monoclonal antibodies directed at different epitopes on the fusion protein of bovine respiratory syncytial virus. J. Infect. Dis..

[bib0140] Vagin A., Teplyakov A. (1997). MOLREP: an automated program for molecular replacement. J. Appl. Cryst..

[bib0145] Verma S., Aitken R. (2012). Somatic hypermutation leads to diversification of the heavy chain immunoglobulin repertoire in cattle. Vet. Immunol. Immunopathol..

[bib0150] Walter T.S., Diprose J.M. (2005). A procedure for setting up high-throughput nanolitre crystallization experiments. Crystallization workflow for initial screening, automated storage, imaging and optimization. Acta Crystallogr. D: Biol. Crystallogr..

[bib0155] Wang F., Ekiert D.C. (2013). Reshaping antibody diversity. Cell.

[bib0160] Waterman D.G., Winter G. (2016). Diffraction-geometry refinement in the DIALS framework. Acta Crystallogr. D: Struct. Biol..

[bib0165] Whyte P. (1994). Identification and Characterisation of Protective B Cell Epitopes on the Fusion Protein of Respiratory Syncytial Virus PhD.

[bib0170] Winter G. (2010). xia2: an expert system for macromolecular crystallography data reduction. J. Appl. Crystallogr..

[bib0175] Zhang B., Chen L. (2017). Protection of calves by a prefusion-stabilized bovine RSV F vaccine. NPJ Vaccin..

