# Supplementary material for: The role of the light chain in the structure and binding activity of two cattle antibodies that neutralize bovine respiratory syncytial virus
Source: Mol Immunol. 2019 Aug;112:123–30. doi: 10.1016/j.molimm.2019.04.026 (PMC6677920; doi:10.1016/j.molimm.2019.04.026)
Supplement: Supplementary file 1 [file mmc1.pdf]

Supplementary data

Figure S1

Sequences of Fab variable domains. CDRs defined according to Kabat E.A., Wu,T.T., Perry,H., Gottesman,K. and Foeller,C. (1991) *Sequences of Proteins of Immunological Interest*, Fifth Edition. NIH Publication No. 91-3242.

(a) VH

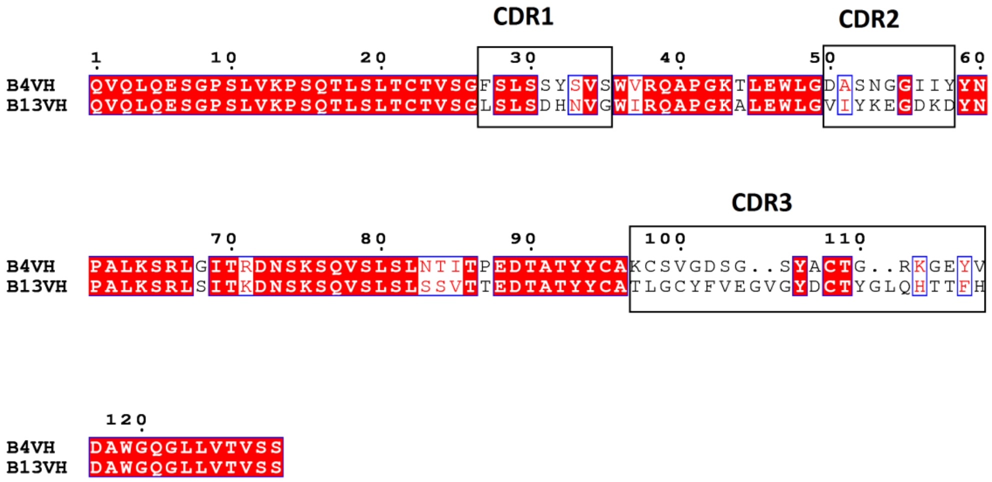

(b) VL

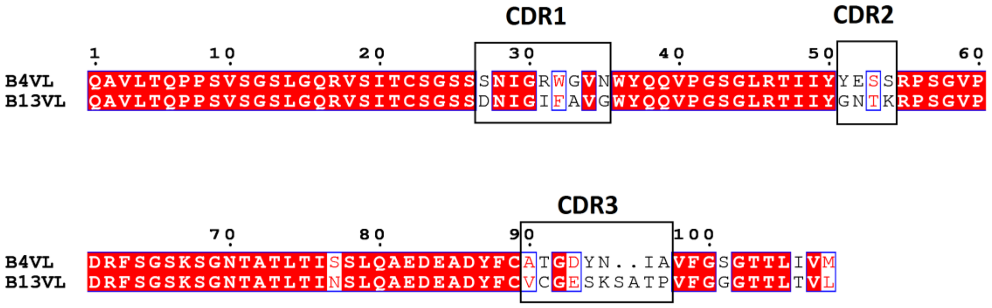

Table S1

Analysis of the variable region interface of the bovine Fabs

	No of residues in interface HC	No of residues in interface LC	Interface area/total surface area HC (Å ^{2})	Interface area/total surface area LC (Å ^{2})
B4	28	28	972 / 6636	970 / 5599
B4*	26	28	838 / 6557	883 / 5645
B13	30	31	917 / 7216	967 / 5692
B13*	31	25	952 / 6912	993 / 5637

Table S2

Buffer conditions for crystallization of bovine Fabs

Fab	Crystallization screen	Crystallization condition
B4	Index, Well F8	0.2 M Ammonium Sulphate<br>25.0% w/v Polyethylene Glycol 3350<br>0.1 M HEPES pH 7.5
B4*	JCSG+, Well A9	20.0% w/v Polyethylene glycol 3350<br>0.2 M Ammonium Chloride
B13	JCSG+, Well C3	20.0% w/v Polyethylene glycol 3350<br>0.2 M Ammonium Nitrate
B13*	Index, Well D11	28.0% w/v Polyethylene Glycol Monomethyl Ether 2000<br>0.1 M bis-Tris pH 6.5
